# Technical note: factors affecting dose distribution in the overlap region of two-segment total body irradiation by helical tomotherapy

**DOI:** 10.1186/s13014-020-01698-x

**Published:** 2020-11-07

**Authors:** HaiYang Wang, JunQi Liu, YiFei Pi, Qi Liu, Yang Mi, XiangXiang Yang, YueXin Guo, RuiTai Fan

**Affiliations:** 1 Department of Radiation Oncology, The First Affiliated Hospital of Zhengzhou University, Zhengzhou, 450000 People’s Republic of China; 2 Department of Radiation Oncology, University of California, San Francisco, CA USA; 3 Department of Marshall Medical Center, The Fifth Affiliated Hospital of Zhengzhou University, Zhengzhou, 450000 People’s Republic of China

**Keywords:** Helical tomotherapy, Gap distance, Field width, Pitch, Total body irradiation

## Abstract

**Objective:**

To assess the effects of various treatment planning parameters to identify the optimal gap distance for precise two-segment total body irradiation (TBI) using helical tomotherapy (HT) with fixed jaw mode.

**Methods and materials:**

Data of a treatment plan for 8 acute leukemia patients (height range: 109–130 cm) were analyzed. All patients underwent total-body computed tomography (CT) with 5-mm slice thickness. A lead wire, placed at 10 cm above the patella, was used to mark the boundary between the two segments. Target volumes and organs at risk were delineated using a Varian Eclipse 10.0 physician’s workstation. Different distances between the lead wire and the boundary of the two targets were used. CT images were transferred to the HT workstation to design the treatment plans, by adjusting parameters, including the field width (FW; 2.5 cm, and 5 cm), pitch (0.287 and 0.430), modulation factor (1.8). The plans were superimposed to analyze the dose distributions in the overlap region when varying target gap distances, FWs, pitches to determine the optimal combinations.

**Results:**

The pitch did not affect the dose distribution in the overlap region. The dose distribution in the overlap region was mostly homogeneous when the target gap distance was equal to the FW. Increased FW diminished the effect of the target gap distance on the heterogeneous index of the overlap region.

**Conclusions:**

In two-segment TBI treatments by HT with Helix mode, a gap distance equal to the FW may achieve optimal dose distribution in the overlap region.

## Background

Total body irradiation (TBI) is a radiation therapy technique that forms an important component of the pretreatment of patients for hematopoietic stem cell or bone marrow transplantation [1–4]. TBI in conjunction with chemotherapy serves as the preparative regimen to avoid immunologic rejection of transplanted donor blood stem cells or bone marrow, by suppressing the immune system of recipients before transplantation. Moreover, it can eradicate residual disease cells in patients and empty space in the spinal cord, thereby increasing the success rate of bone marrow and stem cell transplantation [2, 4–7].

**Fig. 1:**
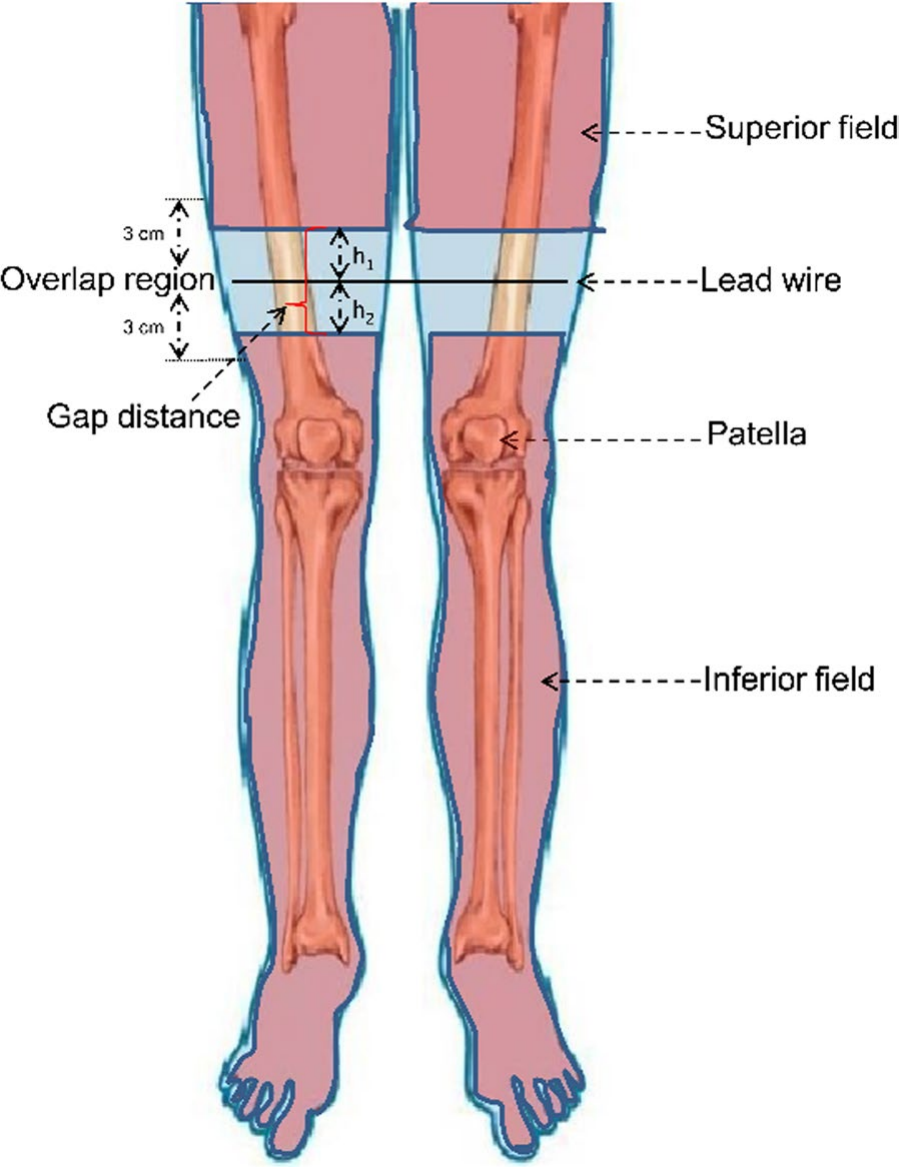
Schematic illustration of total body irradiation in the overlap region. The lead wire was placed at a point 10 cm above the patella. The gap distance contained the distance between the superior field and lead wire (h1) and another distance between the inferior field and lead wire (h2)

Helical tomotherapy (HT) is an intensity modulated radiation therapy (IMRT) applied using megavoltage computed tomography (MVCT), which can deliver radiation in a slice-by-slice pattern, rather than as vertical beams, as generated in linear accelerator (LINAC)-based radiation therapy [4, 8]. HT-based IMRT differs from conventional LINAC-based IMRT in some respects. Firstly, HT delivers a narrow pencil beam modulated by its unique pneumatic multileaf collimator [4]. A rotating gantry delivers radiation while the patient table simultaneously moves. Consequently, it can achieve a highly conformal dose distribution for complex clinical targets while protecting organs at risk (OARs) [7]. Secondly, a large radiation field (135 cm × 40 cm) is preferred for TBI treatment via HT [8]. For a patient less than 135 cm in height, HT can achieve treatment using a single radiation field (i.e., single-segment TBI); however, for patients with a height exceeding 135 cm, TBI is split into a superior field and an inferior field (i.e., two-segment TBI) [9, 10]. The distance between these two fields is defined as the gap distance. The gap distance from the boundary of the superior/inferior fields to the marker line (at a point 10 cm above the patella) may affect the dose distribution of the overlap region, including an area of 3 cm above and below the marker line (Fig. 1). Therefore, using an optimal distance can avoid dosimetric hot or cold spots in the overlap region, reducing the chances of radiation-related complications and treatment failure [11].

The effects of the gap distance in TBI has not been reported previously. Gruen et al. used HT to pretreat pediatric and adult patients prior to stem cell transplantation [10]. They adopted the following treatment parameters: a field width (FW) of 5 cm and a distance of 2 cm from the marker line to both the superior and inferior field boundary (i.e., a gap distance of 4 cm). In another TBI study, Salz et al. used a FW of 5 cm, and a gap distance of 2 cm, by placing the marker line 1 cm from the boundaries of both the superior and inferior fields [12]. However, there is no established standard for parameter configurations. This study evaluated the effects of various parameters (target gap distances, FW, pitch, modulation factor) on the dose distribution in the overlap regions, to establish optimal parameters in HT-TBI.

## Methods

### Patient selection

This retrospective study included 8 patients (6 males and 2 females) with acute leukemia who required TBI pretreatment for bone marrow transplantation. Patient details are shown in Table 1.

**Table 1:** Patient characteristics

Patient no.	Age (years)	Gender	Diagnosis	Body height (cm)	Body weight (kg)
1	8	M	All	125.0	29
2	6	M	AML	109	19.1
3	10	F	All recurrence	122	30.4
4	7	M	ALL	114.5	22.7
5	11	M	AML	128.5	34.1
6	8	F	AML	116	24.8
7	10	M	ALL	130	33
8	9	M	ALL	127.5	28.5

### Immobilization and CT scanning

All patients were immobilized by using a single body board, a head/neck thermoplastic mask, a thermoplastic mask for the abdomen, and a vacuum cushion. Before the CT scan, a lead-wire was placed at 10 cm above the patella, as the marker of the patient’s body surface, to split the radiation field into a superior and an inferior field. Patients then underwent CT (120 kV, SOMATOM Definition AS, Siemens, Erlangen, Germany) scanning from the cranium to the toes, using a slice thickness of 5 mm. These CT image sets were prepared for delineation of target volumes and OARs.

### Contouring and planning

For each patient, the target volumes were delineated using the planning system on the Varian Eclipse physician’s workstation, version 10.0. Superior and inferior field data sets were created using different parameters; these contained the outer body contour (distance of 3 mm to the surface), clinical target volume (CTV), and the OARs (lens, lungs, etc.) [7–9, 12]. The distance between the lower boundary of the superior field and the lead-wire was equal to the distance between the lead wire and the upper boundary of the inferior field (h_1_ = h_2_; Fig. 1). Distances of 0.5 cm, 1 cm, 1.5 cm, 2 cm, 2.5 cm, or 3 cm (h_1_ = h_2_ = 0.5 cm, 1 cm, 1.5 cm, 2 cm, 2.5 cm or 3 cm), resulting in gap distances of 1 cm, 2 cm, 3 cm, 4 cm, 5 cm, and 6 cm, respectively, were used. The overlap region covered an area of 3 cm above and below the lead-wire. The delineated target volumes and CT images were then transferred to the HT planning station for treatment planning.

The prescription dose of 12 Gy was given to each patient in 2-Gy fractions twice a day, on 3 consecutive days [2], at an interval of at least 8 h between fractions. Different plans were designed for different radiation fields. At least 95% of the target area should receive the prescribed dose in the HT plan. Lungs dose were suppressed to an average dose (Dmean) not exceeding 10 Gy and a minimum dose (Dmin) of less than 8 Gy [10]. All plans were finished by two senior physicists using TomoHelical technology. The effects of variation in FW (1, 2.5, and 5 cm) and pitch (0.287, 0.43) were investigated. The modulation factor was constant at 1.8.

### Assessment and statistical analysis

The mean, maximum, and minimum dose as well as the heterogeneous index (HI) were evaluated in the overlap region. The HI was calculated as HI = D_2_/D_98_, where D_2_ was the minimum dose in 2% of the target volume and D_98_ was the minimum dose in 95% of the target volume.

All analyses were performed in SPSS software, version 19.0 (IBM Corp., Armonk, NY). The results are showed as mean ± standard deviation (x ± s). Graphs were drawn in OriginLab 8.0 (OriginLab Corp., Northampton, MA).

## Results

The superior/inferior HT plans with different gap distances (1 cm, 2 cm, 3 cm, 4 cm, 5 cm, and 6 cm) and differences in other parameters (FW and pitch) were superimposed on the physician's workstation. The mean dose, maximum, and minimum dose, and the HI of the overlap region were collected and analyzed in Table 2.

**Table 2:** Mean dose, maximum dose, minimum dose, and heterogeneous index (HI) of the overlap region (= h1 + h2, and h1 = h2)

Other parameters			Target gap distance					
			1 cm	2 cm	3 cm	4 cm	5 cm	6 cm
Pitch = 0.287	FW = 2.5 cm	Mean dose (Gy)	15.04 ± 0.08	13.21 ± 0.13	11.39 ± 0.13	9.58 ± 0.16	7.79 ± 0.13	6.00 ± 0.13
		Max dose (Gy)	18.25 ± 0.20	13.74 ± 0.17	12.85 ± 0.15	12.78 ± 0.21	12.65 ± 0.13	11.69 ± 0.16
		Min dose (Gy)	12.72 ± 0.11	12.67 ± 0.09	9.03 ± 0.19	4.68 ± 0.24	2.13 ± 0.27	1.44 ± 0.15
		HI	1.43 ± 0.20	1.08 ± 0.17	1.42 ± 0.19	2.73 ± 0.24	5.94 ± 0.27	8.12 ± 0.16
	FW = 5 cm	Mean dose (Gy)	19.53 ± 0.27	17.98 ± 0.42	16.34 ± 0.13	14.52 ± 0.26	12.73 ± 0.25	10.99 ± 0.19
		Max dose (Gy)	22.10 ± 0.35	19.82 ± 0.53	17.55 ± 0.15	15.13 ± 0.30	13.02 ± 0.21	12.51 ± 0.19
		Min dose (Gy)	16.32 ± 0.23	15.09 ± 0.24	13.98 ± 0.15	13.10 ± 0.06	12.52 ± 0.28	10.37 ± 0.17
		HI	1.35 ± 0.35	1.31 ± 0.53	1.26 ± 0.15	1.15 ± 0.3	1.04 ± 0.28	1.21 ± 0.19
Pitch = 0.43	FW = 2.5 cm	Mean dose (Gy)	14.96 ± 0.10	13.21 ± 0.16	11.45 ± 0.15	9.52 ± 0.17	7.92 ± 0.22	6.02 ± 0.19
		Max dose (Gy)	18.43 ± 0.33	13.72 ± 0.20	12.65 ± 0.23	12.59 ± 0.22	12.64 ± 0.18	11.55 ± 0.26
		Min dose (Gy)	12.70 ± 0.10	12.63 ± 0.07	9.22 ± 0.28	4.69 ± 0.18	2.26 ± 0.26	1.37 ± 0.23
		HI	1.45 ± 0.33	1.09 ± 0.2	1.37 ± 0.28	2.68 ± 0.22	5.59 ± 0.26	8.43 ± 0.26
	FW = 5 cm	Mean dose (Gy)	19.32 ± 0.31	17.93 ± 0.18	16.27 ± 0.26	14.55 ± 0.32	12.72 ± 0.30	10.95 ± 0.27
		Max dose (Gy)	21.89 ± 0.46	19.78 ± 0.25	17.45 ± 0.36	15.16 ± 0.39	13.04 ± 0.26	12.49 ± 0.17
		Min dose (Gy)	16.17 ± 0.21	15.00 ± 0.24	13.89 ± 0.18	13.07 ± 0.18	12.48 ± 0.32	10.26 ± 0.38
		HI	1.35 ± 0.46	1.32 ± 0.25	1.26 ± 0.36	1.16 ± 0.39	1.04 ± 0.32	1.22 ± 0.38

### Field width of 5 cm

**Fig. 2:**
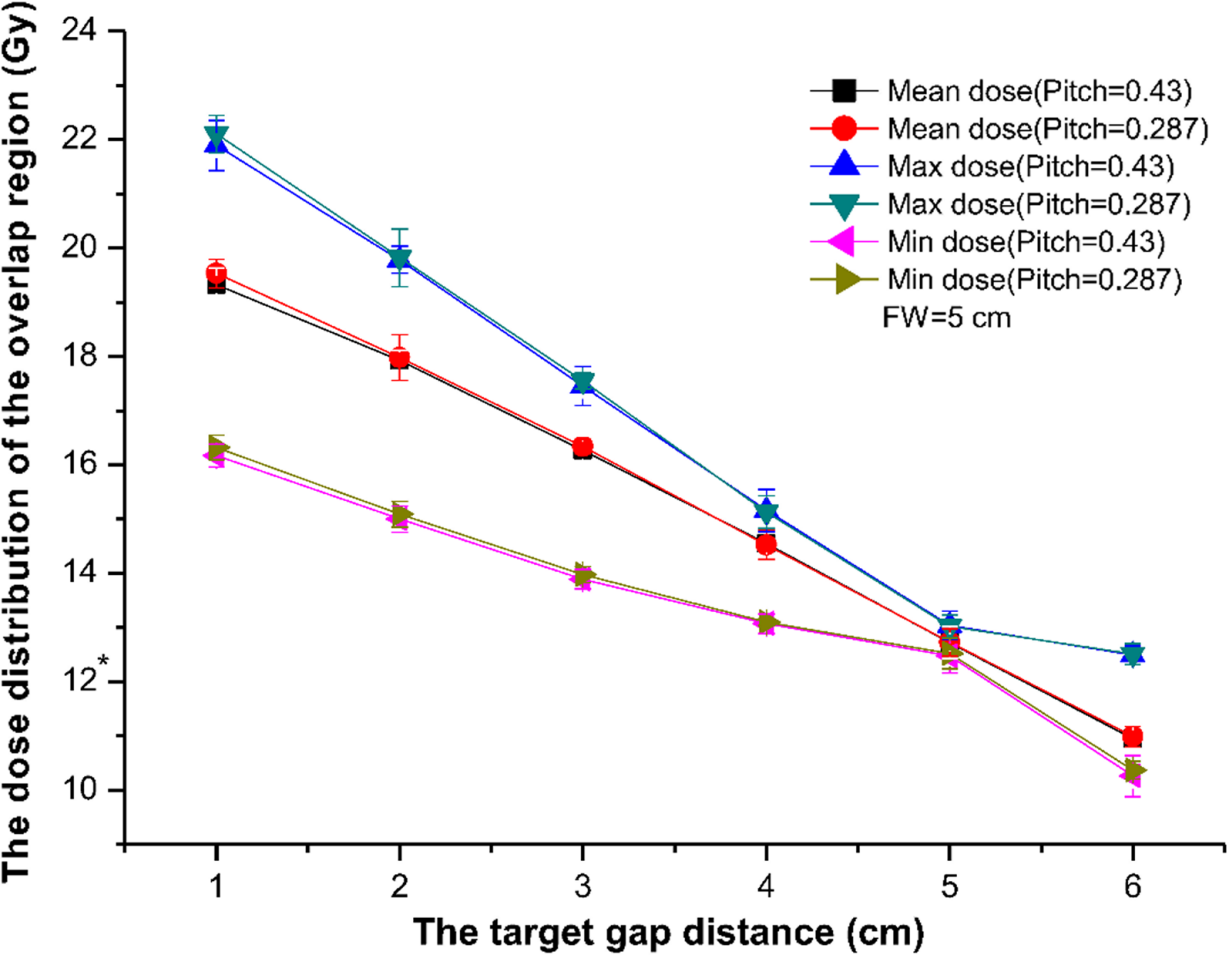
The mean dose, the maximum dose, and the minimum dose of the overlap region varied with different target gap distances. The field width (FW) is 5 cm and pitch are 0.43/0.287. *prescription dose

As shown in Fig. 2, the mean, maximum, and the minimum dose of the overlap region decreased as the gap distance increased, when the FW was 5 cm. Varying pitch (0.43/0.287) did not have an effect on the dose distribution of the overlap region. When the gap distance was 5 cm, the three curves nearly overlapped. All doses at this point were closest to the prescription dose. The minimum dose of the overlap region was higher than the prescription dose when the gap distance was less than 5 cm; the overlap region was therefore a hot spot. In contrast, the size of cold spots increased when the target gap distance exceeded 5 cm. Therefore, a gap distance of 5 cm was optimal for a FW of 5 cm.

### Field width of 2.5

**Fig. 3:**
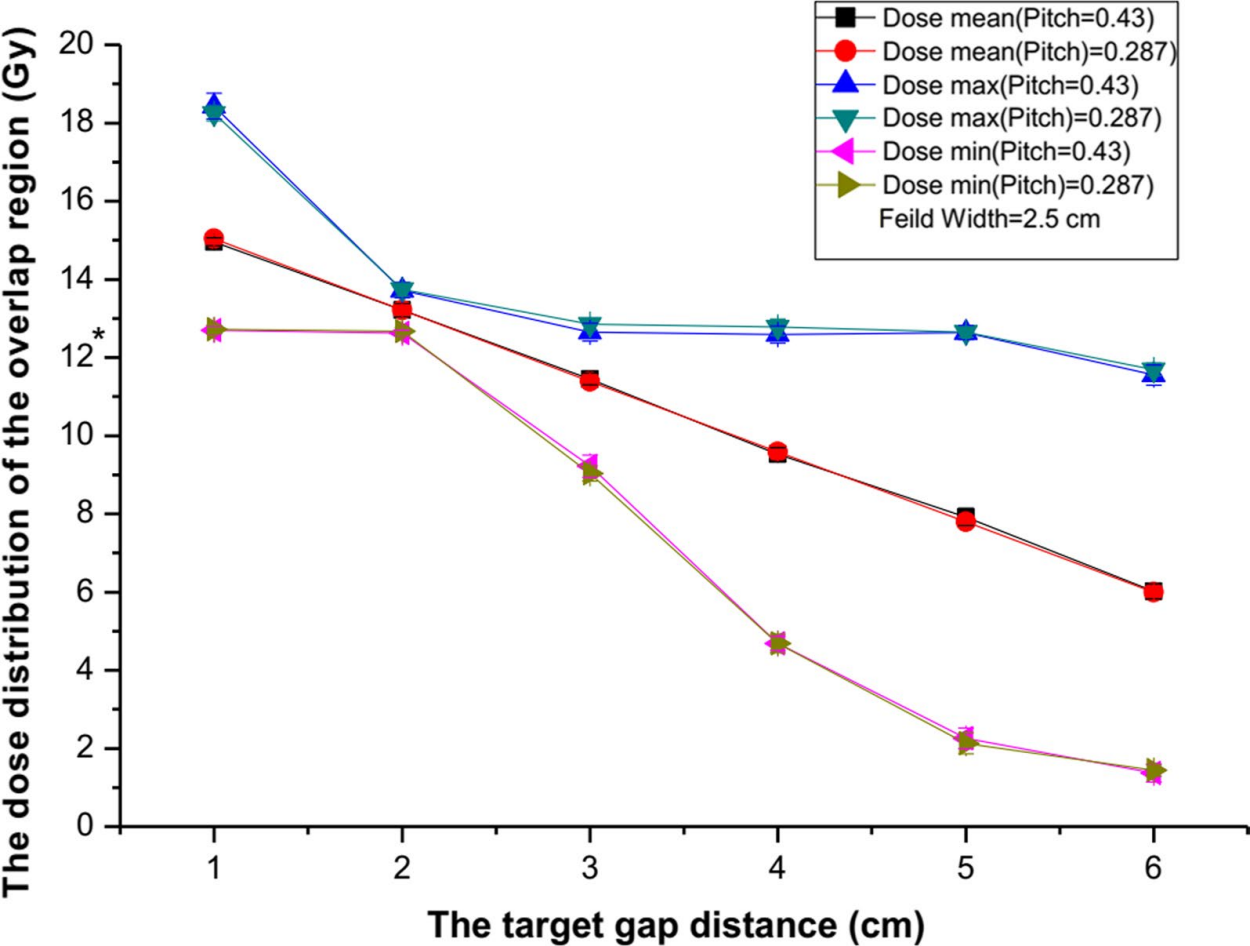
The mean dose, the maximum dose, and the minimum dose of the overlap region varied with the different target gap distances of 2.5 cm. Pitch is 0.43/0.287. *prescription dose

If the FW was 2.5 cm, the optimal gap distance was 2 cm. Similar to the overlap region observed with a FW of 5 cm, there were hot or cold spots if the gap distance was more or less than 2 cm, respectively (Fig. 3a). Pitch did not have an effect on the dose distribution with a FW of 2.5 cm.

### Heterogeneous index

**Fig. 4:**
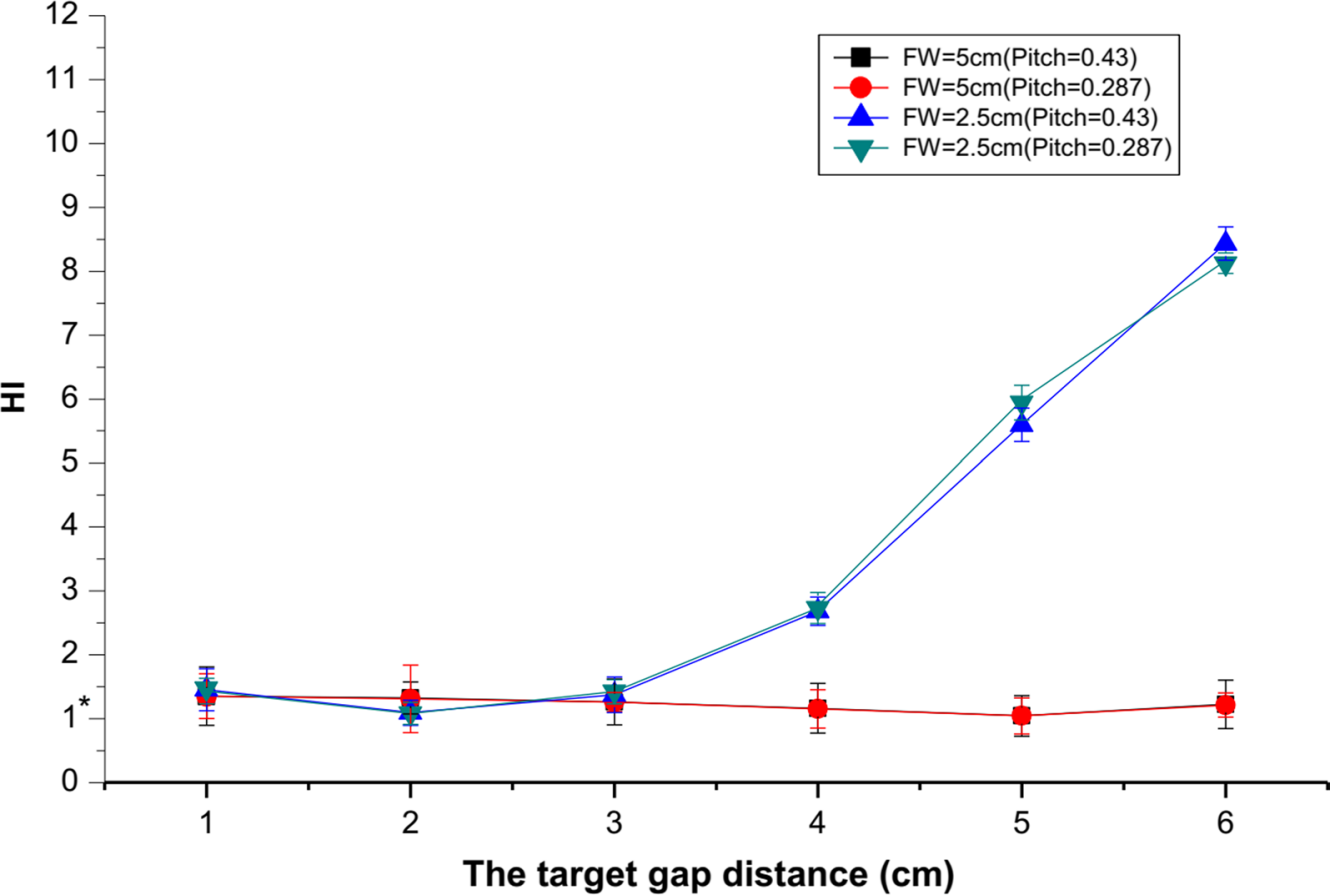
Comparison of heterogeneous index (HI) with different field widths (FW) and pitch in the overlap region for the choice of the optimal target gap distance

The HI is used to describe the dose-distribution heterogeneity in a target volume. An HI of 1.0 means a perfect treatment plan, with homogeneous dose distributions; however, HI values always exceed 1. Pitch values did not affect the quality of the dose distribution in the overlap region according to HI analysis (Fig. 4). HI values were lower (range 1.1–1.5) at varying target gap distances in the overlap region when the FW was set at 5 cm. Notably, the value of HI at a FW of 5 cm was 1.04 ± 0.09 when the target gap distance was 5 cm, indicating an optimal treatment plan with a homogeneous dose distribution in the overlap region. However, the HI value increased gradually when the target gap distance deviated from 5 cm. A gap distance of 2 cm yielded an HI value of 1.09 ± 0.03 when FW was at 2.5 cm.

## Discussion

Various TBI treatment strategies have been widely applied clinically to eliminate malignant cells and prevent graft rejection while reducing the risk of complications and preserving function and quality of life. Some radiosensitive OARs limit the application of high dose radiation, because of radiation-induced complications, such as radioactive pneumonia, radiation nephropathy, or blindness. At present, available data suggest that the dose to the kidney should be limited to 12 Gy to prevent proteinuria, glomerular filtration rate decrease, and other renal inadequacy symptoms from occurring post-radiotherapy [13]. The total TBI dose was 12 Gy (in 6 fractions) in this study, and thus only lens and lungs were considered as critical dose-limiting structures requiring protection [10]. Our center has completed TBI in many patients and have delivered a lower dose to the lens and lungs, resulting in fewer side-effects.

The HT planning system only allows superimposition of treatment plans that were generated on the same CT set. Thus, the 8 patients chosen for this study were about 120 cm tall and actually received single-segment TBI, rather than two-segment TBI. In the study, the delineation of the planning target volume was performed on a set of CT images covering the whole body of the patient. We used “overlap region” to associate plan design. There may thus be a small difference as compared with actual, clinical two-segment TBI; this small error can be reduced by an MVCT scan [8]. In an earlier study, radiation oncologists used two-segment TBI [14]. However, they performed their study with an FW of 5 cm only, while the influence of changes in planning parameters (FW, pitch, etc.) was not investigated. In contrast, we studied and analyzed all planning parameters and therefore present more comprehensive conclusions.

When the treatment plan is designed using different gap distances, the dose to lungs and lens should be reduced as much as possible in the superior field [10]. Hence, even when optimization parameters remained constant, the optimization of radiotherapy treatment planning with various gap distances shows some differences that directly affect the dose distribution in the overlap region, hindering identification of the optimal gap distance. However, we analyzed the mean dose, the maximum dose, the minimum dose, and the HI index in the overlap region when searching for the optimal gap distance. In different treatment plans, the dose in the overlap region was mostly homogeneous when the distance was equal to the FW. Therefore, a small difference in radiotherapy treatment planning will not significantly affect the dose distribution if the optimal target gap distance is used. Keisuke et al. showed that field joint dose distribution calculated by reducing the target volume at the field boundary surface by 2.5 cm (gap distances = 5 cm) with 5 cm FW width was found to be the most robust in the cranial–caudal directions, and utilizing the IMRT technique for the field boundary area could feasibly control the field joint dose distribution [15]. The best dose distribution were also been found in this paper with the same configuration.

The reason for using an overlap region of 6 cm is that the overlap region has to cover the greatest gap distance used in this study. If the overlap region is too small, the dose distribution of target gap distance cannot be shown sufficiently; inversely, if it is too large, the dose distribution between the gap distance and the overlap region will decrease the sensitivity of the test of important indicators, e.g., the mean dose, the maximal dose, the minimal dose, and the HI.

Fan beams of HT plans are modulated by one set of jaws and pneumatic binary multileaf collimators (MLCs). For the HT machine used in this study, there were three jaw options for treatments: 1.0, 2.5, and 5.0 cm. FW of 1 cm was not investigated in this study for the reason that it was too time-consuming to make patients complete treatments. In this study, plans with FW of 2.5 cm showed no obvious improvements concerning dose indexes compared to 5 cm FW plans with suitable FW configurations. We recommend using a gap distance of 5 cm with 5 cm FW for TBI cases. The implications of our findings could also be applied to other cases, such as total skin treatment and multiple metastases.

All HT plans were finished by in Helix mode. Some studies showed potential benefits to improve TBI plan quality in TomoDirect mode: less treatment time and sufficient dose distribution [16]. Optimized gap distance for TomoDirect plans should also be investigated in the future. A third-generation TOMO accelerator has been developed; while the first- and second-generation accelerators adopted a fixed jaw mode, the third generation of TOMO HDA incorporates a dynamic jaw function, which can effectively control the vertical dose drop gradient of the target area. In this study, we used a second-generation TOMO HD, and thus our conclusions are applicable to the first- and second-generation TOMO, but not to the third-generation TOMO HDA. Thus, further studies are needed to confirm these findings in the third-generation TOMO HAD [17]. The leg Diameter will do influence gap distance selection. This study included 8 patients with similar ages and body weight. We did not consider the impact of leg diameter for the lack of enough data. Deeper studies may carry based on more kinds of patients.

## Conclusions

HT is one of the technologies preferred for completing TBI treatment. Two-segment TBI based on HT can be used to prepare taller patients (height > 135 cm) for hematopoietic stem cell or bone marrow transplantation. In the two-segment TBI HT treatment, a gap distance that is identical in size to the FW may achieve the optimal dose distribution in the overlap region.

## Data Availability

The datasets used and/or analyzed during the current study are available from the corresponding author on reasonable request.

## Abbreviations

TBI: Total body irradiation
HT: Helical tomotherapy
CT: Computed tomography
FW: Field width
HI: Heterogeneous index
IMRT: Intensity modulated radiation therapy
MVCT: Megavoltage computed tomography
LINAC: Linear accelerator
OARs: Organs at risk
